# Desmoglein-2 is important for islet function and β-cell survival

**DOI:** 10.1038/s41419-022-05326-2

**Published:** 2022-10-29

**Authors:** Kay K. Myo Min, Darling Rojas-Canales, Daniella Penko, Mark DeNichilo, Michaelia P. Cockshell, Charlie B. Ffrench, Emma J. Thompson, Olof Asplund, Christopher J. Drogemuller, Rashmi B. Prasad, Leif Groop, Shane T. Grey, Helen E. Thomas, Thomas Loudovaris, Thomas W. Kay, My G. Mahoney, Claire F. Jessup, P. Toby Coates, Claudine S. Bonder

**Affiliations:** 1 Centre for Cancer Biology, University of South Australia and SA Pathology, Adelaide, SA Australia; 2 Flinders Renal Laboratory, Renal Unit, Division of Medicine and Critical Care, Southern Adelaide Local Health Network, Flinders Medical Centre, Bedford Park, SA Australia; 3 Adelaide Medical School, University of Adelaide, Adelaide, SA Australia; 4 Central Northern Adelaide Renal and Transplantation Service (CNARTS), Royal Adelaide Hospital, Adelaide, SA Australia; 5 Genomics, Diabetes and Endocrinology, Department of Clinical Sciences, Lund University, Malmö, Sweden; 6 Immunology Department, Garvan Institute of Medical Research, Darlinghurst, NSW Australia; 7 St Vincent’s Institute of Medical Research & the University of Melbourne, Melbourne, VIC Australia; 8 Department of Dermatology and Cutaneous Biology, Thomas Jefferson University, Philadelphia, PA USA; 9 College of Medicine and Public Health, Flinders University, Adelaide, SA Australia

**Keywords:** Mechanisms of disease, Cadherins

## Abstract

Type 1 diabetes is a complex disease characterized by the lack of endogenous insulin secreted from the pancreatic β-cells. Although β-cell targeted autoimmune processes and β-cell dysfunction are known to occur in type 1 diabetes, a complete understanding of the cell-to-cell interactions that support pancreatic function is still lacking. To characterize the pancreatic endocrine compartment, we studied pancreata from healthy adult donors and investigated a single cell surface adhesion molecule, desmoglein-2 (DSG2). Genetically-modified mice lacking *Dsg2* were examined for islet cell mass, insulin production, responses to glucose, susceptibility to a streptozotocin-induced mouse model of hyperglycaemia, and ability to cure diabetes in a syngeneic transplantation model. Herein, we have identified DSG2 as a previously unrecognized adhesion molecule that supports β-cells. Furthermore, we reveal that *DSG2* is within the top 10 percent of all genes expressed by human pancreatic islets and is expressed by the insulin-producing β-cells but not the somatostatin-producing δ-cells. In a *Dsg2* loss-of-function mice (*Dsg2*^lo/lo^), we observed a significant reduction in the number of pancreatic islets and islet size, and consequently, there was less total insulin content per islet cluster. *Dsg2*^lo/lo^ mice also exhibited a reduction in blood vessel barrier integrity, an increased incidence of streptozotocin-induced diabetes, and islets isolated from *Dsg2*^lo/lo^ mice were more susceptible to cytokine-induced β-cell apoptosis. Following transplantation into diabetic mice, islets isolated from *Dsg2*^lo/lo^ mice were less effective than their wildtype counterparts at curing diabetes. In vitro assays using the Beta-TC-6 murine β-cell line suggest that DSG2 supports the actin cytoskeleton as well as the release of cytokines and chemokines. Taken together, our study suggests that DSG2 is an under-appreciated regulator of β-cell function in pancreatic islets and that a better understanding of this adhesion molecule may provide new opportunities to combat type 1 diabetes.

## Introduction

Pancreatic islets are endocrine tissue powerhouses containing an assortment of specialized cells such as the insulin-producing β-cells, the glucagon-producing α-cells, and the somatostatin-producing δ-cells to maintain homeostasis. Dysfunction of pancreatic islets manifests in disease, with the autoimmune destruction of β-cells resulting in the complete insulin deficiency observed in type 1 diabetes (T1D), and broader islet dysfunction resulting in insulin resistance in type 2 diabetes (T2D) [1]. Although significant advances have identified that the execution of glucose-stimulated insulin release by the β-cells is exquisitely regulated [2–4], new knowledge is still required to combat this family of debilitating and deadly diseases.

The 3D arrangement of cells within a pancreatic islet is of immense importance to β-cell function [2, 3] with supportive cell types, such as other endocrine cells and vascular endothelial cells, directing islet development and coordinating responses to glucose [5–8]. The adherens junction proteins, particularly the cadherin family of Ca^2+^-dependent adhesion molecules, are well documented to provide the structural integrity to cells and the intracellular signalling pathways pivotal in islet formation, structure, and function (reviewed in refs. [3, 9–11]). Most relevant to the pancreas, E-cadherin facilitates the initial clustering of β-cells during embryonic development via its link to catenins (α and β) and the actin cytoskeleton, as well as signalling systems to modulate cell viability and insulin production [9, 12–15]. A similar role for N-cadherin has been reported for β-cell survival [14] and insulin secretion [16]. These observations raise the possibility that additional members of the cadherin family may regulate islet function. Interestingly, the desmoglein family of cadherin proteins (DSG1-4) mediates key cell–cell interactions and intracellular signalling in epithelial cells and cardiac myocytes (reviewed in ref. [17]), yet their role in pancreatic function remains completely unknown.

Desmoglein-2 (DSG2), like its other three family members, is a single pass transmembrane cell surface protein that undergoes homotypic and/or heterotypic interactions to form Ca^2+^-dependent adhesive interfaces between adjacent cells [18]. These interactions were first described as adhesive desmosomal complexes and utilized by epithelial cells to tolerate mechanical stress [19]. However, DSG2 is emerging as a unique family member expressed by non-desmosome forming cells, such as embryonic stem cells [20] and vascular endothelial cells (ECs) [21, 22] where it assists with cell survival, proliferation, and neoangiogenesis [22]. When ectopically expressed in the epidermis of transgenic mice, DSG2 enhances signalling pathways and proliferation [23], and its colocalization with the epidermal growth factor receptor augments cellular migration and invasion [24]. Most recently, it has been shown that DSG2 can engage with non-desmosomal cadherins (e.g. E- and N-cadherin) to support cell-cell adhesion [25], harness Rap1 and downstream TGFβ signaling to influence both cell spreading and focal adhesion protein phosphorylation [26] and modulate the production and release of extracellular vesicles from keratinocytes [27, 28]. Important post-translational modification of DSG2, via palmitoylation, regulates the transport of proteins to the plasma membrane [29]. Taken together, these protein transport functions of DSG2 are akin to those performed by pancreatic β-cells and thus raises the possibility that DSG2 may also play a role in metabolic homeostasis.

Herein, we demonstrate that DSG2 is upregulated on pancreatic islet cells, particularly the β-cells (human and murine). Our in vivo work comparing WT and *Dsg2*^lo/lo^ mice suggest that DSG2 is important for islet number, islet size, β-cell survival and glucose-stimulated insulin production. We show that islets harvested from the *Dsg2*^lo/lo^ mice are inferior to their WT counterparts for insulin production, and that they are more susceptible to apoptotic cell death in response to TNFα, IL-1β, and IFNγ. The Beta-TC-6 cells provide further insight into a role for DSG2 in homeostasis of the actin cytoskeleton. Congruent with this, the *Dsg2*^lo/lo^ mice exhibited increased susceptibility to hyperglycaemia and their islets are less effective than their WT counterparts at curing diabetic mice following transplantation.

## Methods

### Patients

Normal pancreas tissue, formalin-fixed and paraffin-embedded, was provided by the Thomas Jefferson University Hospital Pathology Lab or were purchased (Abcam, Cambridge, UK). For islet isolation, healthy human pancreata were obtained with informed consent from next of kin, from heart-beating, brain-dead body donors, with research approval from the HREC committee at St Vincent’s Hospital, Melbourne. Human islets were purified by intraductal perfusion and digestion of the pancreas with collagenase followed by purification using Ficoll density gradients [30]. Purified islets were cultured in Connaught Medical Research Laboratories (CMRL) 1066 medium (Gibco; Thermo Fisher Scientific, Waltham, MA, USA) supplemented with 4% human serum albumin (Australian Red Cross, Melbourne, VIC, Australia), 100 U/ml penicillin, 100 mg/ml streptomycin (Gibco) and 2 mM L-glutamine (Gibco), in a 37 °C, 5% CO_2_ humidified incubator.

### In silico gene expression analysis

#### Microarray dataset

Human pancreatic islets (*n* = 8) were separated by a closed loop method involving collagenase, protease, and density gradient centrifugation as previously described [31]. Approximately 5000 IEQ pure islets were processed for microarray analysis using an Affymetrix HGU133+2 microarray with data normalized using RMA in Partek software (Partek Inc., St Louis, MO, USA) resulting in log_2_ microarray data as described elsewhere [31]. Probeset annotations were downloaded from Affymetrix.

#### RNA sequencing dataset

A second cohort of human pancreatic islets (*n* = 188) as well as fat (*n* = 12), liver (*n* = 12) and muscle (*n* = 12) were obtained through the EXODIAB network from the Nordic Transplantation Program (http://www.nordicislets.org). Isolation of total RNA and gene expression analysis via RNAseq was conducted as described elsewhere in a newly developed tool Islet Gene View (IGW) [32], where resulting counts per million (CPM) values were converted into Fragments Per Kilobase Million (FPKM) values by dividing the transcripts by their effective lengths and multiplying with 1000. Sequencing data is available via the European Genome-phenome Archive under the accession numbers: EGAS00001004042 and EGAS00001004044.

### Animals

Animal experiments were approved by the Animal Ethics Committee of SA Pathology, University of South Australia, or Flinders University. All experiments conformed to the guidelines established by the ‘Australian Code of Practice for the Care and Use of Animals for Scientific Purposes’. All animal experiments were conducted using either DSG2 whole body knockdown (*Dsg2*^lo/lo^ generated via a ‘gene trap’ targeted insertion of a FRT-flanked lacZ-neomycin cassette into intron 1 of the mouse *Dsg2* locus on embryonic stem cells prior to germline transmission and mouse colony generation [22]) or WT controls, all of which were on a C57Bl/6N background. For each experiment, a ‘Power/Sample Size Calculator’ such as G*Power 3.5.1 (Keil University, Germany) was used to determine the minimum number of animals required to obtain statistical significance and mice were randomly allocated to protocols or treatment groups.

### Islet isolation

Pancreatic islets were isolated as previously described [33]. Briefly, 3 ml cold M199 medium (Sigma-Aldrich, St. Louis, MO, USA) containing 0.67 mg collagenase (Liberase TL grade; Roche, Basel, Switzerland) per pancreas was infused into the pancreatic duct in situ, and the surgically excised pancreas was digested at 37 °C for 14–16 min. Islets were purified on a discontinuous Ficoll gradient (GE Healthcare, Chicago, IL, USA). Following extensive washing, islets were cultured free-floating (37 °C, 5% CO_2_) in RPMI (Sigma-Aldrich) supplemented with L-glutamine, penicillin, streptomycin, and 10% foetal calf serum (RPMI-FCS) for up to 4 days. Islets were handpicked for GSIS and apoptosis assays.

### Transmission electron microscopy (TEM)

Samples were fixed overnight at 4 °C in a solution of 4% paraformaldehyde, 1.25% glutaraldehyde in 0.1 M phosphate buffer pH7.2 to which 4% sucrose had been added. They were then washed in phosphate buffer and post fixed in 1% osmium tetroxide for 1 h. The samples were then dehydrated through a series of ethanol washes followed by propylene oxide and epon/araldite resin before being placed in embedding moulds with fresh resin and set in an oven at 60 °C for 48 h. 1 micron survey sections were cut on a Leica UC7 ultramicrotome, mounted on glass slides and stained with 0.05% toluidine blue made up in borax buffer then imaged with a light microscope. When a suitable area was found, 90 nm sections were cut using a diamond knife and collected onto 200 mesh copper grids. Grids were stained with 4% uranyl acetate and lead citrate and examined with a Tecnai G2 spirit 120 kV TEM and digital images recorded with AMT Nanosprint15 MKII and blinded prior to analysis.

### Streptozotocin (STZ) induced diabetes, Intraperitoneal Glucose Tolerance Test (IPGTT), Intravenous Glucose Tolerance Test (IVGTT), Glucose Stimulated Insulin Secretion (GSIS)

STZ induced diabetes and IPGTT experiments were performed as previously described [34] using male mice 8–10 weeks of age. Mice were administered a single dose of 185 mg/kg STZ (Sigma-Aldrich) to induce diabetes. After STZ injection, blood glucose, body weight and animal health were monitored daily and mice that exhibited BGLs of ≥16.0 mmol/L on ≥3 consecutive days were considered diabetic.

IVGTTs were performed using male and female mice at 8–10 weeks of age, after fasting the mice for 6 h, followed by a body weight adjusted intravenous injection of D-glucose (Sigma-Aldrich) at a dose of 1 g/kg body weight per mouse. BGLs were measured at 0, 5, 10, 15, 30, and 60 min post glucose injection.

GSIS: after an overnight incubation, the isolated islets using male and female mice at 8–12 weeks of age of age were treated with either 2 mM or 20 mM glucose-Krebs solutions at 37 °C for 2 h. Secreted insulin concentrations in the supernatants were quantified by Enzyme Linked Immunosorbent Assay (ELISA, high sensitivity insulin ELISA; Crystal Chem, Downer’s Grove, IL, USA). Insulin released from islets in response to low glucose (2 mM glucose) for 1 h, and high glucose (20 mM) for 10 min and 50 min was measured corresponding to basal, 1st and 2nd phase insulin release, respectively. Stimulation indices were calculated as the mean insulin production at high glucose divided by the mean insulin production at low glucose normalized to total protein content.

### Blood Glucose Level (BGL) measurements

Blood glucose concentration measurements for all experiments were determined from tail vein bleeds using a glucometer with blood glucose test strips (Freestyle Optium Neo, Abbott, NSW, Australia).

### Histology and immunofluorescence staining

Human islets were cytospun onto glass slides, fixed with 4% paraformaldehyde (Bio-strategy, VIC, Australia), and membranes permeabilized using 0.25% Triton X100 prior to probing with the guinea pig anti-human/mouse insulin antibody (Abcam) and mouse anti-human DSG2 (clone AF947, R&D Systems, Missouri, MN, USA).

Histological sections of the mouse pancreata from male and female mice (8–12 weeks) or human pancreata were stained with either haematoxylin and eosin to quantify islet numbers and size in mice, or probed with primary antibodies following heat-mediated antigen retrieval in Tris-EDTA buffer (pH 9) or sodium citrate buffer (pH 6). Slides were blocked with 5% normal goat serum in CAS-Block (Thermo Fisher Scientific) or 1% Bovine Serum Albumin (Sigma-Aldrich) for 30–45 min, followed by primary antibody incubation overnight at 4 °C and a secondary antibody incubation the subsequent day for 1 h at RT. All antibody dilutions were in CAS-Block with 5% goat serum. Sections were probed with the following antibodies: guinea pig anti-human/mouse insulin pAb, mouse anti-mouse glucagon mAb, and rat anti-mouse somatostatin mAb (all Abcam), anti-DSG2 mAbs (clone AF947 R&D Systems, and clone 10D2 (gift, James K Wahl III)), rabbit anti-E-cadherin pAb (Cell Signalling Technologies, Danvers, MA, USA) and isotype controls (e.g. IgG (Abcam or R&D Systems) or anti-maltose clone 12B12, gift, James K Wahl III). Secondary antibodies used were goat anti-guinea pig Alexa Fluor 488 or 568, goat anti-mouse Alexa Fluor 555 or 594, goat anti-rabbit Alexa Fluor 488 or goat anti-rat Alexa Fluor 488 (all Life Technologies, Thermo Fisher Scientific). Slides were mounted in Fluoro-Gel mounting medium (PST) and blinded images were taken by fluorescent microscopy (Zeiss, Germany or Nikon, Japan).

Immunohistochemistry (IHC) was performed to label CD31 after heat-mediated antigen retrieval in citric acid buffer (pH 6), and overnight incubation with primary mAb against CD31 (rabbit anti-mouse, Cell Signalling Technologies). The polymer system ADVANCE HRP (Dako Australia Pty Ltd, VIC, Australia) employing DAB as the detection system was used for detection of primary mAb binding. Counterstaining was performed using Mayer’s hematoxylin. The islet circumference was demarked by a dotted line and QuPath used to auto analyse blinded bioimages captured from up to 35 islets from four mice per group.

### mRNA extraction, cDNA synthesis, and quantitative Real-Time Polymerase Chain Reaction (qRT-PCR)

Total RNA was extracted using RNeasy Micro Plus Kits or RNeasy Mini Kits (QIAGEN, Hilden, Germany) as per the manufacturer’s instructions. Conversion of RNA into first strand cDNA (generated from 0.5–1 μg of RNA) was performed by using Superscript III Reverse Transcriptase (Life Technologies; Thermo Fisher Scientific).

Quantification of mRNA levels was carried out using qPCR. *Dsg2* gene expression levels were validated using primers designed to span mouse *Dsg2* intron/exon border between exons 14 and 15 (F- 5′AACGAAGCCGTAAGGACAAG 3′ R- 5′ GCCGCTTTCTCTGTGAAGTA 3′) using Primer Blast (NIH, MD, USA), and purchased from GeneWorks (Hindmarsh, SA, AUS). qPCR amplification was performed using QuantitectTM SYBR Green master mix (QIAGEN) on a Rotor-Gene thermocycler (QIAGEN) with reaction parameters: 15 min at 95 °C, then cycling of 10 s at 95 °C, 20 s at 55 °C and 30 s at 72 °C; for 45 cycles followed by a melt phase. Data was obtained and analyzed using Rotor-Gene Analysis Software version 6 (QIAGEN). All samples were run in triplicate. Relative gene expression levels were calculated using the comparative quantitation method normalized to the housekeeping gene hypoxanthine-guanine phosphoribosyltransferase (*Hprt1*) expression (F-5′CCCAGCGTCGTGATTAGCG3′ R-5′GCACACAGAGGGCCACAATG3′).

### Intravital microscopy

To measure in vivo barrier integrity, intravital microscopy of the mouse ear vasculature was performed [35]. Mice (6–8 weeks old) were sedated using an intra-peritoneal injection of a 10 mg/ml ketamine/xylazine mixture at a dosage of 1 μl per gram. The mouse ear was then placed over a raised platform and mounted under a glass coverslip in preparation for imaging. Prior to imaging, the mouse was allowed to rest for 30 min to reduce any potential inflammation that may have resulted from the manual handling. To visualise the vasculature, 100 μl of 10 mg/ml FITC-dextran (70 kDa) was injected intravenously via an intra-orbital injection. The mouse ear was then positioned under a 20× objective within a heated chamber of an LSM 710 two-photon microscope (Zeiss). The FITC-Dextran was excited using a tuneable Mai Tai Ti:Sapphire multiphoton laser (Spectra-Physics, Santa Clara, USA) and external non-descanned detectors were used to capture the fluorescence signal. A stack of 3 images over a range of 10 μm was then acquired every 5 min for 15 min. Image analysis was undertaken using a macro written for use within ImageJ [36]. As all images were in colour, the green channel was split out and then a median filter with a radius of 2.0 pixels was employed to reduce noise. A fluorescence threshold was then manually applied by the user to the time zero image, with subsequent images in the series then using the threshold values from the time zero image. Image analysis then determined the percentage area covered by the threshold region for 5–6 mice in each group with data blinded until analysis was complete.

### Cytokine induced apoptosis assay

Isolated islets from male and female mice (8–12 weeks of age) were subject to a 72 h incubation with a cytokine mixture of: 120 ng/ml IFNγ, 200 ng/ml TNFα, 0.5 ng/ml IL-1β (R&D Systems) prior to digestion with 0.5 ml Accutase (used neat; Sigma-Aldrich), to generate a single cell population and staining with Annexin V-APC (apoptotic marker; BD Biosciences) and Propidium Iodide (PI; cell death marker; Life Technologies) and analyzed on the BD Acurri C6v flow cytometer (BD Biosciences) with data analysis using FCS Express 4 Flow Cytometry: Research Edition (De Novo Software, CA, USA).

### Flow cytometric analysis of DSG2 cell surface protein expression in human islets

Isolated human islets were digested with Accutase (Sigma-Aldrich) to generate a single cell population, which were then stained with a viability dye 7-AAD (Life Technologies), an isotype control (purified mouse IgG1, BD Biosciences, New Jersey, USA), mouse anti-human DSG2 (clone 6D8, Life Technologies) for 30 min at 4 °C. Cells were washed twice then stained with a secondary antibody; goat anti-mouse-DyLight 650 (Abcam) as well as a β-cell marker Newport Green (Invitrogen; Thermo Fisher Scientific) for 30 min at 4 °C. Cells were washed and then resuspended in FACS fix (1% formaldehyde, 20 g/L glucose, 5 mM sodium azide in PBS) and analyzed on the BD Accuri C6 flow cytometer (BD Biosciences). Data was further analyzed using FCS Express 4 Flow Cytometry: Research Edition (De Novo Software).

### A marginal mass islet transplantation under the kidney capsule

Male mice (8–12 weeks of age) were rendered diabetic by a single dose intraperitoneal injection of 185 mg/kg STZ (Sigma-Aldrich) in citrate buffer (0.1 M tri-Sodium Citrate Buffer pH 4.5, made fresh on the day of injection), all injections were performed within 10 min of solubilization of STZ. Diabetes was confirmed by two consecutive blood glucose readings >16.6 mM. Diabetic mice were transplanted with a marginal mass (200 islets) of cultured islets (isolated from mice 7–16 weeks of age) under the kidney capsule as previously described [34]. Blood glucose levels were monitored 5–7 days per week for a period of 35 days. Cure of diabetes was defined as the first day of two consecutive non‐fasted blood glucose readings of <11.1 mM with no subsequent reversion to hyperglycaemia. Mice that were defined as cured then underwent intraperitoneal glucose tolerance test. Mice were fasted for 4 h and given 2 mg/kg glucose by intraperitoneal injection (Sigma-Aldrich). Tail vein blood samples were taken prior to injection and at 15, 30, 60, and 120 min post‐injection. Blood samples (blinded) were analysed for glucose using a glucometer (Accu-Check Performa, Roche Diabetes Care, Mannheim, Germany).

### Murine Beta-TC-6 cells and culture

Beta-TC-6 cells were purchased from the American Type Culture Collection (ATCC, VA, USA), confirmed mycoplasma negative (MycoAlert, Lonza, Basel, Switzerland) and maintained in DMEM media (Gibco, Thermo Fisher Scientific) supplemented with 15% FCS (HyClone, Logan, UT, USA) and 5% GlutaMax (Gibco) in 5% CO_2_ at 37 °C.

### Small interfering RNA transient knockdown of *Dsg2*

Transient silencing of mouse DSG2 expression on the surface of Beta-TC-6 cells was achieved by treating cells with 10 nM of DSG2-targeting 27mer small interfering RNA (siRNA) duplexes (Origene, Rockville, MD, USA) delivered using the Lipofectamine RNAiMAX transfection reagent (Invitrogen, Carlsbad, CA, USA). As a control, cells were also treated with 10 nM of the universal non-silencing siRNA duplex (Origene). Knockdown efficiency was routinely assessed via qPCR at 48 h post transfection.

### Phalloidin immunofluorescence

Beta-TC-6 cells with and without *Dsg2* knockdown were seeded into 24 well plates (Falcon, Corning) containing coverslips that had been pre-coated with 1 mg/ml fibronectin (Roche, Basel, Switzerland) and 72 h later the culture media was aspirated, coverslips washed with PBS and fixed with 4% paraformaldehyde (VWR International, Radnor, PA, USA) for 10 min at RT. Cells were washed and permeabilised with 0.1% Triton X-100 for 10 min at RT, then stained with rhodamine phalloidin (1:1000, Life Technologies) and DAPI (1:2000, Sigma-Aldrich) and incubated for 1 h at RT in the dark. Coverslips were washed and mounted onto glass slides using Fluoro-Gel mounting medium (ProSci Tech, Thuringowa Central, QLD, Australia) and left to cure for 24 h at RT in the dark. Immunofluorescence images were taken on the LSM 800 Confocal Microscope (Zeiss) and processed using the Zen 2011 (Zeiss) and ImageJ Fiji software. A minimum of 30 cells for four independent experiments were measured (total 120 cells). Fluorescence intensity of cell borders was determined by measuring average pixel intensity using the line scan function (lines of the same length and width) of ImageJ. Data was extracted from ImageJ and the area under the curve was calculated using GraphPad PRISM 8.0 (GraphPad Software, San Diego, CA).

### Protein arrays

Beta-TC-6 cells with and without *Dsg2* knockdown were seeded into 6 well plates (Falcon). After 48 h, the media was aspirated, and cells were either treated without or with TNFα (100 ng/ml, Life Technologies) for a further 24 h. Conditioned media was then collected and centrifuged at 1500 rpm for 5 min, the supernatant was separated and stored at −80 °C until use. Cells were lysed directly in the well with lysis buffer (provided and pre-prepared with protease inhibitors). Lysates were collected and clarified by centrifugation (13,000 rpm, 8 min, 4 °C). Protein concentration was determined using a Pierce™ BCA protein assay kit (Thermo Fisher Scientific) according to the manufacturer’s instructions, and the protein concentration was measured at 540 nm using the FLUOstar Omega Microplate Spectrophotometer (BMG Labtech, Mornington, VIC, Australia). The cytokine and phospho-receptor tyrosine kinase (RTK) profile of Beta-TC-6 cells (± siDSG2 and ± TNFα treatment) was assessed using the Proteome Profiler Mouse Array Kits (R&D Systems) according to manufacturer’s instructions. The membrane was visualised using the LAS-4000 (FujiFilm, Tokyo, Japan). Pixel density quantification of the dots on the membrane was performed using ImageJ Fiji software.

### Cell cycle analysis

Beta-TC-6 cells, without and with *Dsg2* knockdown, were seeded into 12 well plates (Falcon) and 48 h after knockdown the cells were synchronised using 0.5% FCS (HyClone) for 8 h. Normal (preferred) media was restored after synchronisation and cells were left to incubate overnight prior to being fixed overnight at −20 °C in 70% ethanol, washed twice in ice-cold PBS then 0.25% Triton X-100 (in PBS). Cells were resuspended in staining solution (Propidium Iodide (25 μg/ml), RNase A (40 μg/ml) and PBS) and incubated for >2 h at RT in the dark prior to analysis on the Cytoflex flow cytometer (Beckman Coulter Life Sciences, NSW, Australia). Cell cycle distribution was analysed using FCS express 6 (De Novo Software).

### Statistical Analysis

Unless otherwise stated, all results are presented as mean ± SEM from at least three independent experiments. When data sets were not normally distributed, nonparametric statistical tests were used. Statistical analyses comparing two groups with each other were performed with a two-tailed T-test using 95% confidence intervals. Statistical analyses comparing three groups or multiple parameters were conducted by two-way analysis of variance (ANOVA) with the Bonferroni post hoc test using 95% confidence intervals. All statistical analyses and preparation of graphs were performed with GraphPad PRISM 8.0 (GraphPad Software) and at all opportunities samples were blinded prior to analysis. * indicates results with *p* < 0.05, **p < 0.01, ***p < 0.001.

## Results

### Desmoglein-2 is expressed in pancreatic islets

**Fig. 1:**
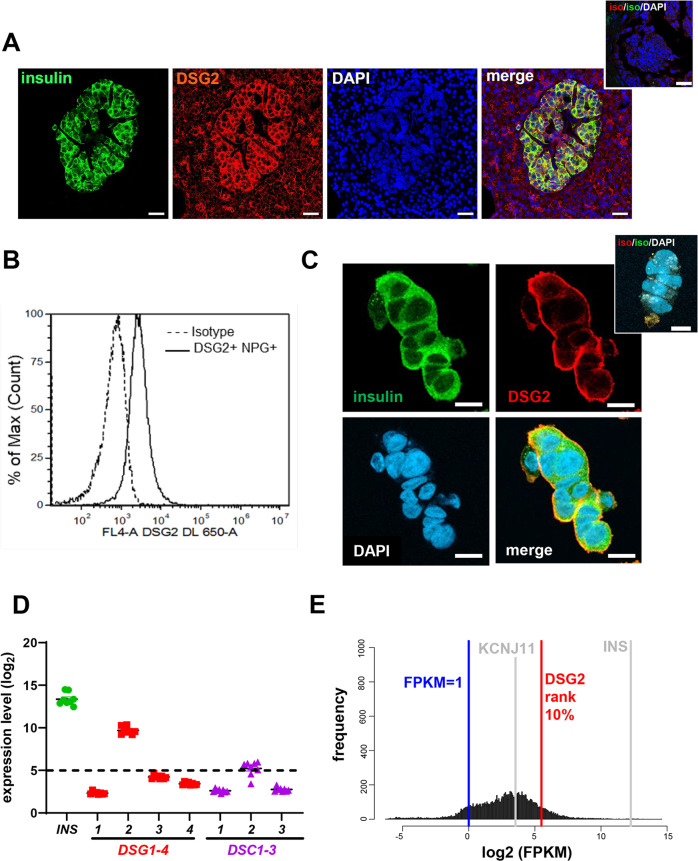
DSG2 protein and gene expression by human islets. **A** Immunofluorescence confocal microscopy of human pancreas from a healthy body donor stained for insulin (green), DSG2 (red), and nuclei (blue). Scale bar = 20 μm. Insert top right is representative of isotype control stains. **B** Surface expression of DSG2 by flow cytometric analysis on freshly isolated human islet cells from healthy donors labelled with Newport Green (NPG) dye identifying β-cells, isotype control (dotted line), and DSG2 (solid line); with all single cells gated from a live population (7-AAD). **C** Immunofluorescence microscopy of partly digested human islets from a healthy donor stained for β-cells by labelling for insulin (green), DSG2 (red), and nuclei (blue). Scale bar = 10 μm. Insert top right is representative of isotype control stains. **D** Microarray gene expression of insulin (*INS*, green), desmogleins *(DSG1-4*, red), and desmocollins *(DSC1-3*, purple) in isolated islet preparations from 9 healthy human body donors. Data represented as the average log2 expression ± SEM with a threshold cut off of 5. **E** Complete RNA sequencing data from 188 human islets expressed as log2 FPKM (Fragments Per Kilobase Million, value of 1 noted in blue line) with ranked expression of *DSG2* (red line) compared to insulin (*INS*, grey line) and potassium channel (*KCNJ1*, grey line).

The role for DSG2 in the pancreatic islet is currently unexplored. Here, we tested for DSG2 protein using immunofluorescence microscopy of formalin-fixed paraffin-embedded (FFPE) sections from healthy human donors. As shown in Fig. 1A, when insulin is used to positively identify pancreatic β-cells, DSG2 staining is clearly identifiable on the pancreatic endocrine cells, and to a lower level in the exocrine tissue. To further validate DSG2 as a cell surface-expressed protein, we assessed DSG2 expression on digested healthy human islets from body donors by flow cytometry. Using Newport Green, a zinc probe used to identify pancreatic β-cells [37], we confirmed that DSG2 is expressed on the cell surface of β-cells (Fig. 1B). Similarly, immunocytochemistry of human islets demonstrated DSG2 positive staining of the insulin-containing β-cells (Fig. 1C).

As DSG2 is one of four desmogleins (DSG1-4), we undertook a retrospective in silico gene expression analysis of eight healthy human islet preparations [31] and observed *DSG2* to be the only desmoglein family member highly expressed by these cells (Fig. 1D). Given that the canonical function of desmogleins is to interact with either desmogleins or desmocollins to form adhesion complexes called desmosomes [19], we also investigated the gene expression of the three desmocollins (DSC1-3) and observed only borderline detectable expression of *DSC2* (Fig. 1D). In the absence of other desmogleins and desmocollins, it is tempting to speculate that DSG2 engages in homotypic interactions. However, expression of E-cadherin by islet cells, and its close proximity to DSG2 (Supplementary Fig. S1) suggests that these two cadherin proteins could interact and supports documentation of this occurring on other cell types [25, 38, 39].

Interestingly, a comparative analysis of *DSG2* expression in a second independent dataset of human islets [32] suggests that it is ranked in the top 10% of all islet genes and sits between the highly expressed insulin (*INS*) and a well-known β-cell gene, the potassium voltage-gated channel subfamily J member 11 gene (*KCNJ11*) [40], which is ranked at 40% of all islet genes expressed (Fig. 1E). Together, these data suggest that DSG2 is a solely represented desmoglein family member in human pancreatic β-cells.

**Fig. 2:**
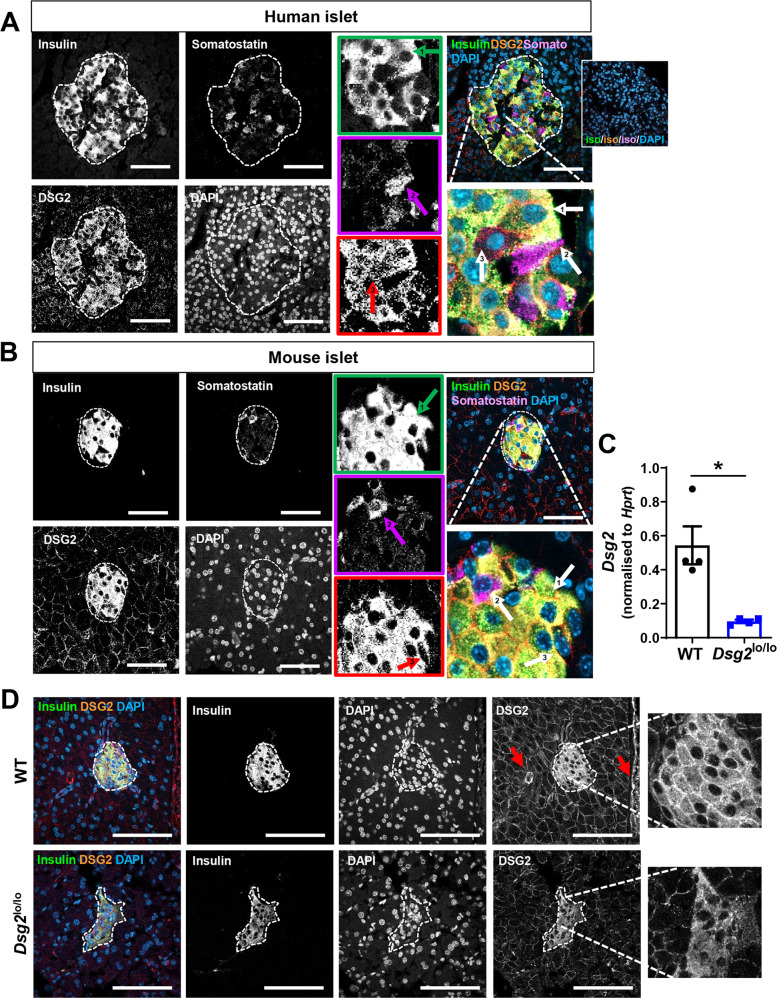
DSG2 expression by select cell types in the islets of humans and mice. Immunofluorescence confocal microscopy of **A** human and **B** mouse islets stained for insulin (green), DSG2 (red), somatostatin (magenta), and nuclei (DAPI, blue). Insets with arrow labelled ‘1’ indicating insulin-positive cells, arrow labelled ‘2’ indicating somatostatin positive cells, and cells with neither insulin nor somatostatin staining indicated with arrow labelled ‘3’. Scale bar = 50 μm. Insert top right is representative of isotype control stains. **C** Mouse pancreata were isolated from wildtype (WT, black circles) and *Dsg2*^lo/lo^ (blue squares) mice and *Dsg2* gene expression determined via qRT-PCR. Data are expressed as mean ± SEM relative to housekeeper gene (*Hprt1*), *n* = 4 mice per group, *p < 0.05 vs WT. **D** Immunofluorescence confocal microscopy of pancreas sections from WT and *Dsg2*^lo/lo^ mice stained for insulin (green), DSG2 (red), and nuclei (blue). Red arrow indicating blood vessels. Scale bar = 50 μm.

To investigate whether DSG2 is restricted to the insulin-producing β-cells or more broadly expressed within the pancreatic islet cluster, sections of human pancreas were stained for DSG2 (red) together with insulin (for β-cells, green) and somatostatin (for δ-cells, magenta) leaving the remainder (e.g. glucagon-producing α-cells) identifiable as those negative for both insulin and somatostatin within the islet cluster. Figure 2A demonstrates DSG2 co-staining with insulin but not somatostatin. Interestingly, Fig. 2A also demonstrates DSG2 expression by islet cells that were negative for both insulin and somatostatin, and likely to be the glucagon-producing α-cells. Similar results were observed in pancreas sections of C57Bl/6 mice with DSG2 detectable on both β-cells and likely α-cells, but not δ-cells within islet clusters (Fig. 2B).

To examine a potential role for DSG2 in pancreatic islet function, we compared wildtype (WT) mice to a whole body *Dsg2* loss-of-function strain of mice (*Dsg2*^lo/lo^) previously reported by us to exhibit significant reduction in *Dsg2* expression in the heart and blood vasculature, but with normal viability and fertility [22]. First, we measured *Dsg2* gene expression in islets harvested from the pancreata of the two murine variants by quantitative real-time PCR with Fig. 2C showing a significant reduction in *Dsg2* expression in the islets from *Dsg2*^lo/lo^ mice when compared to WT controls. Consistent with this, immunofluorescence staining of murine pancreata suggest that DSG2 protein expression is reduced in the *Dsg2*^lo/lo^ mice and possibly also displaced from the cellular junctions (Fig. 2D). Notably, Fig. 2D also demonstrates DSG2 positive blood vessels in the pancreas of the WT mouse (demarked by red arrow).

### *Dsg2*^lo/lo^ mice have smaller pancreatic islets

**Fig. 3:**
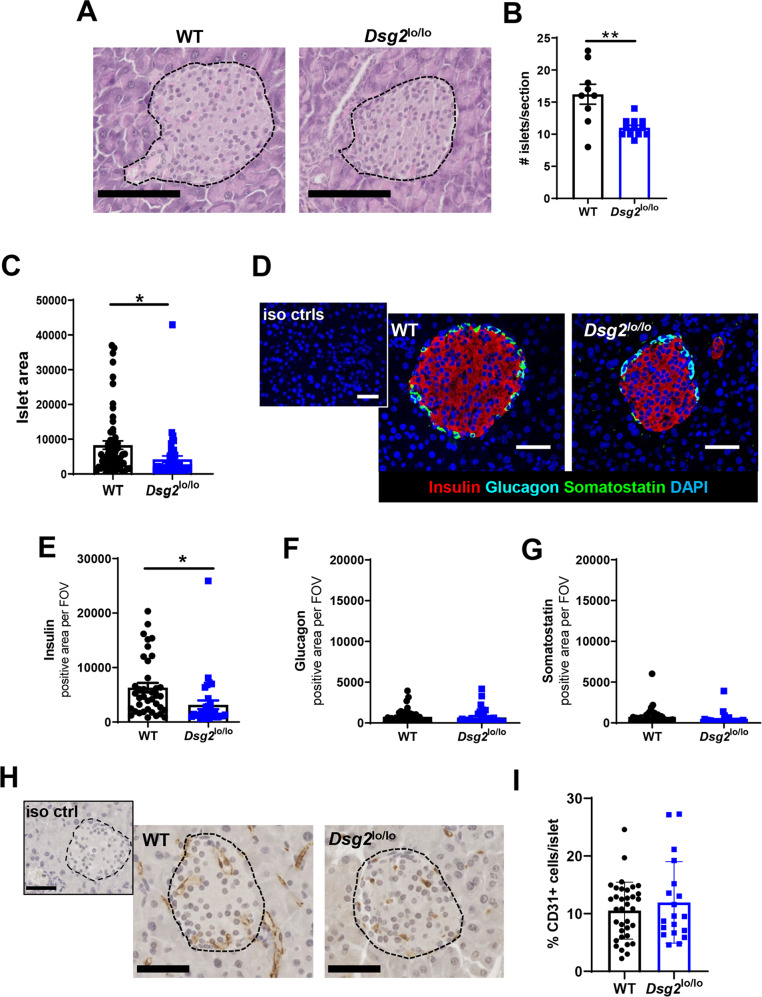
Pancreatic islets in *Dsg2*^lo/lo^ mice compared to wild-type counterparts. **A** Immunohistochemistry of pancreata harvested from WT and *Dsg2*^lo/lo^ mice stained with haematoxylin and eosin to identify islet clusters (black dotted outline) within the exocrine tissue. Scale bar = 100 µm. **B** Numbers of islets quantified from three entire sections across the organ for WT (*n* = 3 mice) and *Dsg2*^lo/lo^ (*n* = 4 mice). Data are expressed as mean ± SEM, **p < 0.01 vs WT. **C** Islet area determined using ImageJ and presented in arbitrary units for the 60 islets assessed from 3 WT mice and 47 islets assessed from 4 *Dsg2*^lo/lo^ mice. Data are expressed as mean ± SEM, *p < 0.05 vs WT. **D** Representative images of immunofluorescence staining on pancreas sections from WT and *Dsg2*^lo/lo^ mice to identify insulin producing β-cells (red), glucagon-producing α-cells (blue), and somatostatin-producing δ-cells (green). Insert top left is representative of isotype control stains. Scale bar = 50 μm, fluorescence staining quantified as pixel intensity for insulin (**E**), glucagon (**F**), and somatostatin (**G**). Data are expressed as mean pixel intensity ± SEM, for the 38 islets assessed from 3 WT mice and 33 islets assessed from 4 *Dsg2*^lo/lo^ mice, *p < 0.05 vs WT. **H** Representative images of immunohistochemistry staining on pancreas sections from WT and *Dsg2*^lo/lo^ mice to identify blood vessels (CD31+). Scale bar = 100 μm. Insert top left is representative of isotype control stains. **I** % CD31 + vessels per islet quantified for the 35 islets assessed from 4 WT mice and 19 islets assessed from 4 *Dsg2*^lo/lo^ mice, data are expressed as mean ± SEM.

To investigate the potential impact of reduced DSG2 in islets, we stained FFPE mouse pancreatic tissue sections with hematoxylin and eosin to identify islet clusters (Fig. 3A). The number of islets were counted from three geographically distinct sections spanning across the pancreas (i.e. head to tail), and suggest that compared WT mice, *Dsg2*^lo/lo^ mice have significantly fewer islets throughout their pancreas (Fig. 3B). We also observed that the *Dsg2*^lo/lo^ islets were significantly smaller in size compared to the WT controls (Fig. 3C). To compare the islet architecture between the WT and *Dsg2*^lo/lo^ mice we stained the sections for insulin (β-cells, red), glucagon (α-cells, blue) and somatostatin (δ-cells, green) (Fig. 3D). While a significant reduction in detectable insulin was observed in the islets of *Dsg2*^lo/lo^ mice versus WT controls (Fig. 3E, attributable to smaller islet size), no discernible difference was observed in the expression levels of glucagon or somatostatin (Fig. 3F, G).

Given the important and intimate relationship between β-cells and vascular ECs in the islet [4, 41] as well as a documented role for DSG2 in angiogenesis [21, 22], we examined the vasculature within the islets of WT and *Dsg2*^*lo/lo*^ mice. Figure 3H demonstrates CD31 stained vasculature in the mouse pancreata from both the WT and *Dsg2*^*lo/lo*^ mice with the percentage of CD31+ECs relative to the total number of cells within each islet cluster similar between the two groups (Fig. 3I).

**Fig. 4:**
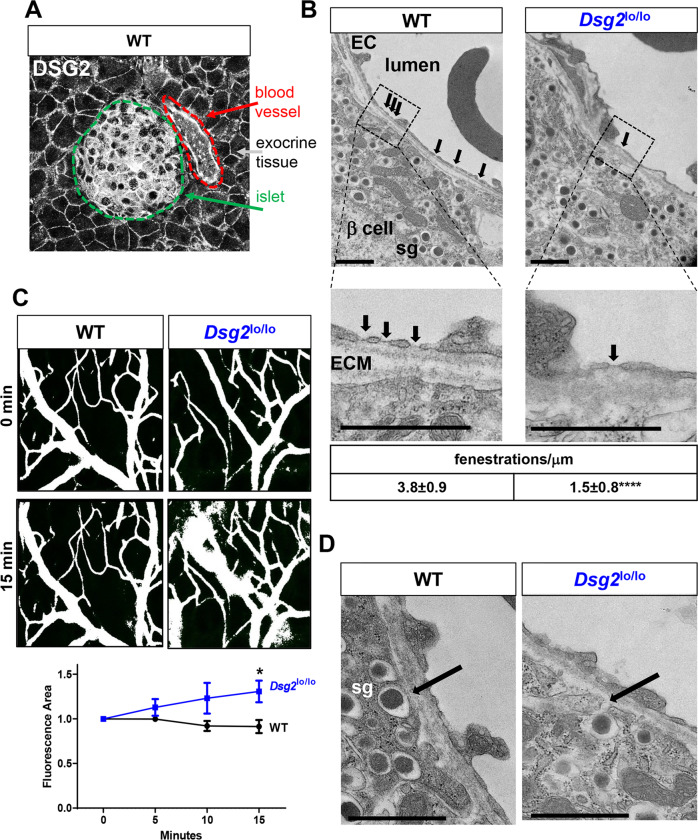
Ultrastructural analysis of pancreatic islets and vasculature in WT and *Dsg2*^lo/lo^ mice. **A** Immunofluorescence confocal microscopy of a WT mouse pancreatic islet stained for DSG2 with DSG2+ pancreatic islet (demarked in green) and DSG2+ blood vessel (demarked in red). **B** Transmission electron microscopy (TEM) of pancreatic islets in WT and *Dsg2*^lo/lo^ mice showing the vasculature endothelial cells (EC) with the lumen on one side and the β-cell on the other. Arrows indicate the EC fenestrations and the insert below shows the sieve plates of fenestrae counted per μm length of vessel lining from *n* = 6–16 islet-associated vessels from 2 mice per group. ****p < 0.0001 vs WT, scale bar = 1 μm. **C** Anaesthetised mice (WT and *Dsg2*^lo/lo^) were injected i.v. with 70 kDa FITC-Dextran prior to intravital 2-photon microscopy of the ear. Snapshots of 0 and 15 min time points were quantified via mean normalised fluorescence of Dextran signal ± SEM (*n* = 5–6 mice), *p < 0.05 vs WT at 15 min. **D** arrows identifying the membranes encasing the insulin-containing granules with their typical electron-dense core. Scale bar = 1 μm.

Having detected DSG2 expression by various cells within the pancreas, including cells within the islet, the blood vasculature, and the surrounding exocrine tissue (Fig. 4A) we next used enhanced microscopy to investigate cell architecture in greater detail. Transmission electron microscopy (TEM) suggests that vascular function may be compromised in the *Dsg2*^lo/lo^ mice as islet-associated ECs exhibited fewer fenestrations per distance of vascular bed in (Fig. 4B). To investigate this further, intravital microscopy was used to compare the barrier integrity of blood vessels within the ears of live (anesthetised) *Dsg*2^lo/lo^ and WT mice using a 70 kDa FITC-Dextran tracer which is reportedly too large to pass through the EC barrier under basal conditions [35]. Figure 4C demonstrates that over the course of 15 min, significant leakage of the 70 kDa FITC Dextran into the interstitial space occurs in the ears of the *Dsg2*^lo/lo^ mice, but not the control WT mice. Notably, while the vascular beds of the ear and the pancreas are distinct, these results support the TEM images of EC barrier integrity being reliant, at least in some capacity, on DSG2. Also observed, but not reliably quantifiable in this format, is a suggestion of compromised vesicular membranes that contain and transport the insulin granules in the *Dsg2*^lo/lo^ mice (Fig. 4D).

### DSG2 loss impacts insulin release and apoptosis at the level of the islet

**Fig. 5:**
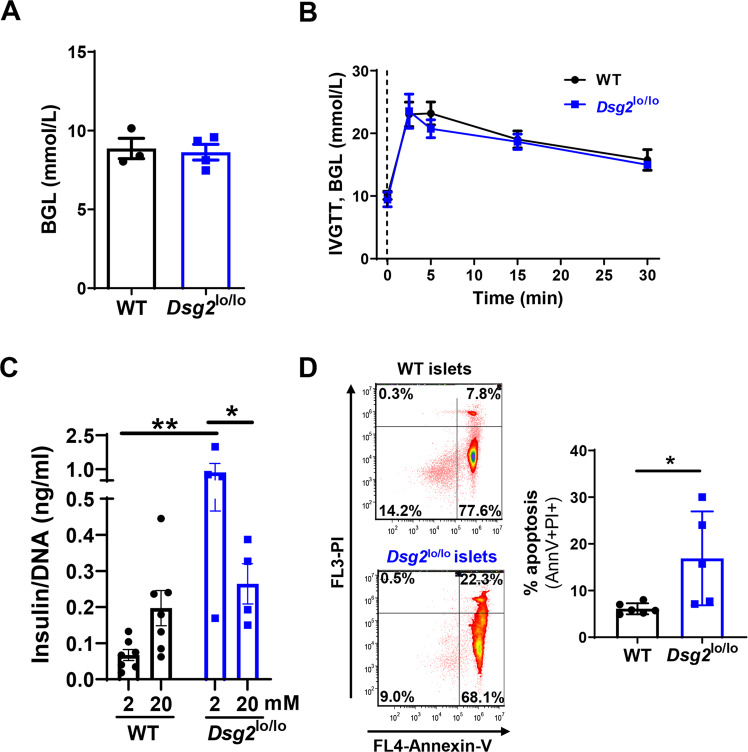
Islet function in *Dsg2*^lo/lo^ and wildtype mice. **A** Baseline blood glucose levels (BGL) in WT and *Dsg2*^lo/lo^ mice. Results are mean ± SEM, *n* = 3–4 mice per group. **B** Glucose tolerance in WT and *Dsg2*^lo/lo^ mice following i.v. injection of 1 g/kg glucose with BGLs measured at 0 (dotted line), 2.5, 5, 15, and 30 min post injection. Data are expressed as mean ± SEM from *n* = 7–9 individual mice per group. **C** Islets isolated from WT or *Dsg2*^lo/lo^ mice tested for glucose-stimulated insulin release at low glucose (2 mM) and then high glucose (20 mM) for 1 h, represented as mean ± SEM of insulin release to DNA, *n* = 4–7 mice per group. **D** Cytokine induced apoptosis of islets isolated from WT and *Dsg2*^lo/lo^ mice. Islets were exposed to TNFα, IL-1β and IFNγ for 72 h prior to staining for with Annexin V and propidium iodide (PI) to assess cell death, *n* = 4–6 mice per group and 3 separate experiments, *p < 0.05 vs WT.

To begin to assess a potential role for DSG2 in glucose metabolism, we examined the baseline blood glucose levels (BGL) in fasted WT and *Dsg2*^lo/lo^ mice and observed no difference (Fig. 5A). Next, we tested glucose clearance in vivo using an intravenous glucose tolerance test (IVGTT) with WT and *Dsg2*^lo/lo^ mice showing similar BGL responses (Fig. 5B).

Using islets isolated from WT and *Dsg2*^lo/lo^ mice, we next assessed glucose stimulated insulin secretion (GSIS) in response to low (2 mM) and high (20 mM) glucose. As expected, healthy islets from WT mice exhibit elevated insulin production in response to high glucose versus low glucose (stimulation index of 5.49) (Fig. 5C). In contrast, islets from *Dsg2*^lo/lo^ mice exhibited the highest basal insulin secretion when exposed to low glucose (0.860 ± 0.394 ng/ml compared to 0.067 ± 0.015 ng/ml in WT islets) and this was not elevated in response to the higher dose of 20 mM glucose (*Dsg2*^lo/lo^ 0.264 ± 0.055 ng/ml in high glucose compared to 0.860 ± 0.394 ng/ml in low glucose).

To investigate whether DSG2 supports islet survival, we challenged isolated islets with the cocktail of pro-inflammatory cytokines implicated in the development of type 1 diabetes; namely TNFα, IL-1β, and IFNγ [42, 43]. Figure 5D shows that following 72 h exposure to TNFα, IL1β, and IFNγ, islets isolated from *Dsg2*^lo/lo^ mice exhibited increased apoptotic cell death as determined by Annexin V^+^/Propidium Iodide^+^ cells. These results suggest that DSG2 plays a protective role in islet cell survival.

### *Dsg2*^lo/lo^ mice are more susceptible to streptozotocin-induced diabetes

**Fig. 6:**
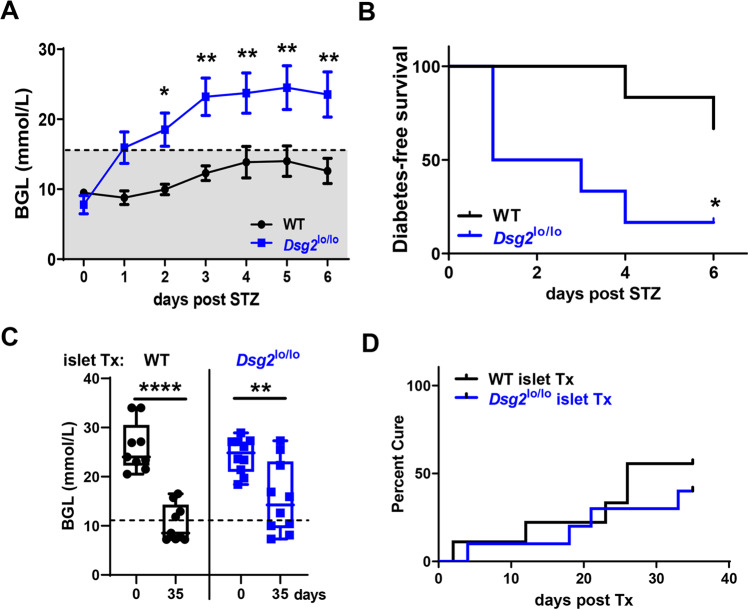
Comparison of STZ-induced diabetes in WT and *Dsg2*^lo/lo^ mice and response of diabetic WT mice to islet transplantation. **A** WT and *Dsg2*^lo/lo^ mice administered STZ (185 mg/kg) were monitored daily for BGLs. A BGL ≥ 16 mmol/L (black dotted line) indicates the diabetic cut off value with the grey shaded box indicating a normal BGL range. Results are mean ± SEM, *n* = 8–9 mice per group, *p < 0.05 & **p < 0.01 vs WT. Area under the curve quantified and presented as mean ± SEM, *n* = 8–9 mice per group, **p < 0.01 vs WT. **B** From A, percentage of mice that became diabetic over time, *p < 0.05 vs WT. **C** Diabetic C57Bl6/N control (WT, *n* = 9) mice were transplanted with marginal islet mass of 200 islets harvested from WT (*n* = 5) or *Dsg2*^lo/lo^ (*n* = 4) mice under the kidney capsule. BGLs in individual mice were recorded daily and up to 35 days post-transplantation. **p < 0.01 vs day 0 BGL, ****p < 0.0001 vs day 0 BGL. **D** From **C**, percentage cure of diabetic mice transplanted with marginal mass of islets displayed as Kaplan–Meier curve, where two consecutive readings of ≤11.1 mmol/L was considered a cured mouse.

To further examine a protective role for DSG2 in β-cell function, we challenged the mice with streptozotocin (STZ), an alkylating agent that targets the insulin-producing β-cells in the pancreas, thus mimicking β-cell loss and hyperglycemia characteristic of type 1 diabetes [44, 45]. Figure 6A demonstrates that when administered with 185 mg/kg of STZ, the *Dsg2*^lo/lo^ mice elevated their BGLs within 24 h to ~16 mmol/L and this continued to rise significantly compared to their WT controls. When assessed as a percentage of ‘diabetes-free survival’ we observed that while 83% of *Dsg2*^lo/lo^ mice became diabetic, only 33% of wildtype animals became diabetic by 6 days post-STZ injection (Fig. 6B). No significant changes in total body mass were observed in either group over the course of the experiment (data not shown).

Next, we compared the ability of islets isolated from WT or *Dsg2*^lo/lo^ mice to cure diabetic mice in a marginal mass transplantation model. Here, male WT mice were rendered diabetic by a single dose of 185 mg/kg STZ prior to 200 islets (harvested from either WT or *Dsg2*^*lo/lo*^ mice) being transplanted under the kidney capsule. Daily BGLs were recorded over 35 consecutive days post-transplant (Supplementary Fig. S2A) with results suggesting enhanced glycaemic control in the mice transplanted with WT islets (Fig. 6C; BGL readings at day 35 for WT islet Tx = 10.5 ± 1.2 mmol/l and for *Dsg2*^lo/lo^ islet Tx = 15.6 ± 2.3 mmol/l, notably a trend but not significantly different). When assessed as the percentage ‘cured’ by day 35, the WT islets conferred a 55% cure rate (5 out of 9) while the *Dsg2*^lo/lo^ islets cured only 40% of the mice (4 out of 10) (Fig. 6D); a trend for improved glycaemic control by the WT islets that warrants further investigation. Notably, while the average fasting BGL of the mice ‘cured’ by *Dsg2*^lo/lo^ islets was significantly higher at 12.8 ± 1.2 mmol/l than that of the WT islets with 8.7 ± 0.7 mmol/l, both groups demonstrated glucose tolerance in response to an i.p. injection of 2 mg/kg glucose (Supplementary Fig. S2B). Together, these results suggest that DSG2 may play a protective role during STZ-induced β-cell death in vivo.

### A role for DSG2 in β-cell cytoskeleton architecture and protein production

**Fig. 7:**
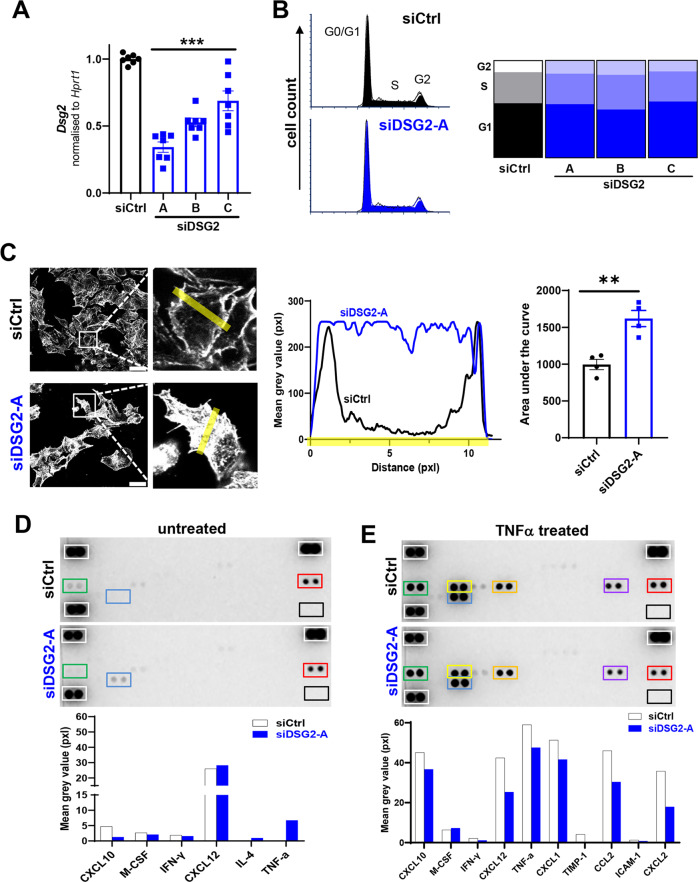
Cell cycle distribution, F-actin regulation and cytokine production by Beta-TC-6 cells without and with *Dsg2* knockdown. **A** Representative qRT-PCR showing *Dsg2* gene expression for siCtrl (black) and siDSG2 (A-C, blue) groups normalised to housekeeper *Hprt1*, *n* = 7 independent experiments, ***p < 0.001. **B** Cell cycle distribution (G0/G1, S or G2 phase) of Beta-TC-6 cells without (siCtrl, black) and with *Dsg2* knockdown (siDSG2, blue) using flow cytometry PI staining, *n* = 3 independent experiments. **C** Representative immunofluorescence image of Phalloidin-labelled filamentous actin in Beta-TC-6 cells without (siCtrl, black) and with *Dsg2* knockdown (siDSG2-A, blue). Yellow rectangle highlights the area of interest which was used to calculate the mean grey value (pixels) across the cell from border to border. The mean grey value for siCtrl (black) and siDSG2-A (blue) was converted to area under the curve (AUC), *n* = 4 independent experiments, **p < 0.01. **D** Cytokine/chemokine array of supernatants harvested from Beta-TC-6 cells without (siCtrl) or with *Dsg2* knockdown (siDSG2-A), *n* = 1 experiment. Mean grey value of duplicate dots was calculated and summarised as a bar graph below. White box = positive control, black box = negative control, green box = CXCL10, blue box = TNF-alpha, red box = CXCL12. **E** Cytokine/chemokine array of supernatants harvested from Beta-TC-6 cells without (siCtrl) or with *Dsg2* knockdown (siDSG2-A) following TNFα treatment (100 ng/ml, 24 h), *n* = 1 experiment. For detectable proteins, mean grey value of duplicate dots was calculated and graphed. White box = positive control, black box = negative control, green box = CXCL10, yellow box = CXCL1, blue box = TNFα, orange box = CCL2, purple box = CXCL2, red box = CXCL12.

To investigate a role for DSG2 in β-cell function, we turned to a reliable β-cell line, the murine Beta-TC-6 cells. Following 48 h knockdown of *Dsg2* via three separate DSG2-targeting siRNAs (Fig. 7A) we performed a cell cycle analysis and observed that loss of DSG2 does not influence the phases of cell cycle (G0/G1, S, and G2) (Fig. 7B).

As actin filaments of the cytoskeleton are important components of cellular dynamics and function, we next investigated whether loss of DSG2 affected actin assembly in Beta-TC-6 cells. Using the aforementioned siRNA knockdown approach, we examined F-actin (via Phalloidin staining) and observed that the actin filament distribution differed with loss of DSG2. For example, Fig. 7C shows that control cells contain actin filaments along the periphery of the cells, while the siDSG2 Beta-TC-6 cells contain actin across the entire cell. Quantitation of Phalloidin fluorescence intensities (illustrated via the opaque yellow box overlay the enlarged images of Fig. 7C) support this observation with control cell F-actin contained within the termini of short bundles at the cell edge, whilst in the siDSG2 cells actin is present across the entire cell surface, a feature captured by area under the curve (Fig. 7C).

Finally, we investigated whether DSG2 knockdown altered the release of cytokines and chemokines using a protein profile array. Supernatants harvested from Beta-TC-6 cells, without or with siDSG2 for 48 h, revealed only low level production of CXCL10, M-CSF, IFNγ, IL-4, and TNFα and modest production of CXCL12 (Fig. 7D). Surprisingly, loss of DSG2 decreased CXCL10 but increased TNFα in the supernatant. In similar experiments, we tested the cells in response to 100 ng/ml TNFα stimulation for 24 h. As expected, both groups responded to TNFα treatment with increase production of CXCL10, M-CSF, CXCL12, CXCL1, TIMP-1, CCL2, ICAM-1, and CXCL2 (Fig. 7E). Notably, siDSG2 cells released less CXCL12, CXCL1, TIMP-1, CCL2, and CXCL2 than the controls. Cell lysates from the aforementioned experiments were also examined for receptor tyrosine kinase (RTK) protein levels via proteome array. Notably, VEGFR3 was the only RTK detectable in the array and it did not change in response to siDSG2 (Supplementary Fig. S3).

## Discussion

Herein we present new knowledge of a desmosomal cadherin, DSG2, being uniquely expressed on the surface of pancreatic islet cells in humans and mice, including insulin-producing pancreatic β-cells and glucagon-producing α-cells, but not somatostatin-producing δ-cells. Surprisingly, we show that *DSG2* is ranked within the top 10% of all genes expressed in the human islet. When comparing WT and *Dsg2*^lo/lo^ mice, our results suggest that DSG2 is important for islet number, islet size and, consequently, glucose-stimulated insulin production. We show that islets harvested from the *Dsg2*^lo/lo^ mice are inferior to their WT counterparts for insulin production and that they are more susceptible to apoptotic cell death in response to TNFα, IL-1β and IFNγ. Consistent with this, the *Dsg2*^lo/lo^ mice exhibited increased susceptibility to STZ-induced hyperglycaemia and their islets were potentially less effective than their WT counterparts at curing diabetic mice following transplantation.

Connectivity between islet cells (hormone-producing as well as vascular ECs) is paramount for circulating blood glucose levels to maintain vital organ function (e.g. cardiovascular and renal [46]) [7] and is achieved via specialized communication networks that extend to other cells and to the extracellular matrix (reviewed in [3, 4, 10, 47]). Intercellular junctions include ‘adherens junctions’ (facilitated via cadherin proteins) that control the adhesion of endocrine cells during selected developmental stages and organisation of different cells within the islets [12, 48]. The extracellular domain of cadherins mediate homotypic adhesion with neighbouring cells while the intracellular domains are linked to the actin cytoskeleton via catenins (α and β) and participate in intracellular signalling systems [10]. Parnaud and colleagues elegantly showed that E-cadherin and N-cadherin (but not P-cadherin) are expressed by human β-cells [14] and that they mediate β-cell survival [14] as well as glucose-stimulated insulin release [16]. Herein we demonstrate co-localization of DSG2 with E-cadherin in pancreatic islets which is consistent with similar observations in intestinal epithelial cells [25, 39]. Whether DSG2 acts alone to formulate cell-to-cell contacts, a role similar to that undertaken by E-cadherin and N-cadherin, is yet to be determined. Reduced expression of DSG2 (via siRNA in the murine cell line Beta-TC-6 cells) disrupted the location of filamentous actin which supports documentation of DSG2 stabilizing F-actin in endothelial cells [22]. Modification of the F-actin network is key to islet cell function as it mediates insulin secretion [49, 50] and results here suggest that DSG2 is protective with loss of DSG2 in the Beta-TC-6 cells increasing the release of TNFα, a known pathogenic and proinflammatory cytokine implicated in diabetes [43]. Why loss of DSG2 suppresses the release of CXCL10 in the Beta-TC-6 cells is not entirely clear as type 1 diabetics are reported to have elevated levels of serum CXCL10 [51], but it may reflect advanced disease as Shigihara and colleagues reported that serum CXCL10 levels decrease immediately following disease onset [52]. In response to exogenous TNFα for 24 h, siDSG2 Beta-TC-6 cells also demonstrated a striking reduction in the release of chemokines CXCL12, CCL2, and CXCL2. Notably, CXCL12 was the most abundant protein produced by the Beta-TC-6 cells (without and with TNFα treatment) and is known to protect and preserve the function of β-cells in the pancreatic islet with crucial roles in β-cell development, survival, regeneration, and immune regulation (reviewed in ref. [53]). Chemokines such as CXCL12, CCL2, and CXCL2 are also vital for vascular development, regulating angiogenesis, and stabilizing the vascular network [54]; all features crucial to maintaining islet function [4, 33]. Tissue Inhibitor of Metalloproteinase-1 (TIMP-1) release by siDSG2 Beta-TC-6 cells was also reduced when compared to control cells, and with a documented pro-survival role in pancreatic islets [55], this data further supports our contention that DSG2 is protective. We also postulate that the post-translational modification of DSG2 via palmitoylation of cysteine residues 635 and 637 (which regulates protein transport to the plasma membrane and the components of the endocytic pathway [27, 28]), may also be important for factors, including chemokine-containing secretory vesicles and insulin-containing granules, to fuse with the plasma membrane for release. Unfortunately, further investigation into the role of DSG2 in human β-cells is currently limited by the absence of a reliable human β-cell line to manipulate DSG2 levels.

DSG2 may also be involved in the intimate connection between β-cells and the vasculature. Pancreatic islets are highly vascularized and receive 10% of the pancreatic blood flow despite comprising only 1–2% of the tissue mass (reviewed in ref. [4]). The bidirectional communication between β-cells and ECs supports not only insulin gene expression and secretion, but also β-cell survival, EC proliferation, and angiogenesis [4, 33, 56]. Currently, DSG2 has limited (or not readily detectable) expression on the broader EC population, with documentation of DSG2 on human endothelial progenitor cells [22], human vasculature in some normal and cancerous tissues [22], human bone marrow vasculature [57], human skin microvascular ECs [58] and high endothelial venules in mouse lymphoid organs [22]. Our identification of DSG2 on WT murine pancreatic vasculature is consistent with our previous report of CD31 + ECs in the pancreas of *Dsg2*^lo/lo^ mice exhibiting regions of increased junctional hypertrophy and an undulating luminal surface [22]. Herein, our TEM of the pancreata in *Dsg2*^lo/lo^ mice further support vascular malformation with compromised fenestrations of the vascular bed. Endothelial fenestrae are transcellular pores within capillary walls that congregate and serve as a diaphragm to regulate the passing of pancreatic hormones between islet cells and the blood circulatory system [59]. Integrins and the extracellular matrix (ECM) are paramount for the development and maintenance of these structures, with fibronectin activating cytoskeletal regulators and remodelling actin [60, 61]. Local integrin activation in β-cells also targets insulin secretion to the islet capillaries [62]. With new intravital data and previous publications by us and others demonstrating that loss of DSG2 in ECs interrupts association with integrin-β_8_, placement of F-actin and VE-cadherin causing loss of barrier integrity [21, 22], it is our contention that deregulated islet function in *Dsg2*^lo/lo^ mice may also be due to a compromised vascular system within the pancreas.

Our data also suggest a protective role for DSG2 in β-cells with islets harvested from *Dsg2*^lo/lo^ mice exhibiting heightened sensitivity to cytokine-induced apoptosis; an observation consistent with ectoptic expression of DSG2 in keratinocytes increasing resistance to anoikis [23]. Similarly, DSG2 knockdown studies in the Beta-TC-6 cells demonstrated an increase in the pro-inflammatory cytokine TNFα and a reduction in the pro-survival chemokine CXCL12 [53]. While the role of DSG2 in influencing β-cell survival and insulin production within the pancreas still remains unclear (and currently limited by the lack of appropriate tools), key β-cell survival signals (i.e. PI3K/Akt, MAPK/ERK, STATs and NFκB (reviewed in [63]) have been linked with DSG2 in other cell types [23]. Finally, with the ProteinPredict program of ExPASy suggesting that DSG2 contains over 13 protein kinase C (PKC)-target motifs in the cytoplasmic domain alone [64], and that epidermal growth factor treatment of A431 epithelial cells induces tyrosine phosphorylation of DSG2 [65], there is much more to uncover about this solitary desmosomal protein on β-cells in metabolic homeostasis.

In conclusion, this study provides novel insights into the function and survival of insulin-producing β-cells and reveals an underappreciated role for the desmosomal cadherin protein DSG2. Our observations support a coordinated regulation of cadherin-mediated adhesion complexes, together with extracellular signalling cues ([62, 66, 67] and reviewed in ref. [2]), for glycaemic control and provide new ‘actionable’ knowledge on the development of diabetes.

## Supplementary information

Supplemental Material

Reproducibility checklist

## Data Availability

All data analyzed during this study are included in the published article (and its online supplementary files) with related references indicating the original resource. No new resources were generated during the current study.
