# Supplementary material for: Desmoglein-2 is important for islet function and β-cell survival
Source: Cell Death Dis. 2022 Oct 29;13(10):911. doi: 10.1038/s41419-022-05326-2 (PMC9617887; doi:10.1038/s41419-022-05326-2)
Supplement: Supplementary file 1 — Supplemental Material [file 41419_2022_5326_MOESM1_ESM.pdf]

Desmoglein-2 is important for islet function and β-cell survival

Kay K. Myo Min[1], Darling Rojas-Canales[2], Daniella Penko[3,4], Mark DeNichilo[1], Michaelia P Cockshell[1], Charlie B. Ffrench[1], Emma J. Thompson[1], Olof Asplund[5], Christopher J. Drogemuller[3,4], Rashmi B. Prasad[5], Leif Groop[5], Shane T. Grey[6], Helen E. Thomas[7], Thomas Loudovaris[7], Thomas W. Kay[7], My G. Mahoney[8], Claire F. Jessup[3,9], P. Toby Coates[3,4] and Claudine S. Bonder[1,3].

1. Centre for Cancer Biology, University of South Australia and SA Pathology, Adelaide, SA, AUSTRALIA
2. Flinders Renal Laboratory, Renal Unit, Division of Medicine and Critical Care, Southern Adelaide Local Health Network, Flinders Medical Centre, Bedford Park, SA, AUSTRALIA
3. Adelaide Medical School, University of Adelaide, Adelaide, SA, AUSTRALIA
4. Central Northern Adelaide Renal and Transplantation Service (CNARTS), Royal Adelaide Hospital, Adelaide, SA, AUSTRALIA
5. Genomics, Diabetes and Endocrinology, Department of Clinical Sciences, Lund University, Malmö, SWEDEN
6. Immunology Department, Garvan Institute of Medical Research, Darlinghurst, NSW, AUSTRALIA
7. St Vincent's Institute of Medical Research & the University of Melbourne, Melbourne, Vic, AUSTRALIA
8. Department of Dermatology and Cutaneous Biology, Thomas Jefferson University, Philadelphia, Pennsylvania, USA.
9. College of Medicine and Public Health, Flinders University, Adelaide, SA, AUSTRALIA.

Corresponding author:
Claudine S. Bonder
Centre for Cancer Biology, University of South Australia & SA Pathology, Adelaide, SA, AUSTRALIA; Ph: + 61 8 83027833,
email:claudine.bonder@unisa.edu.au

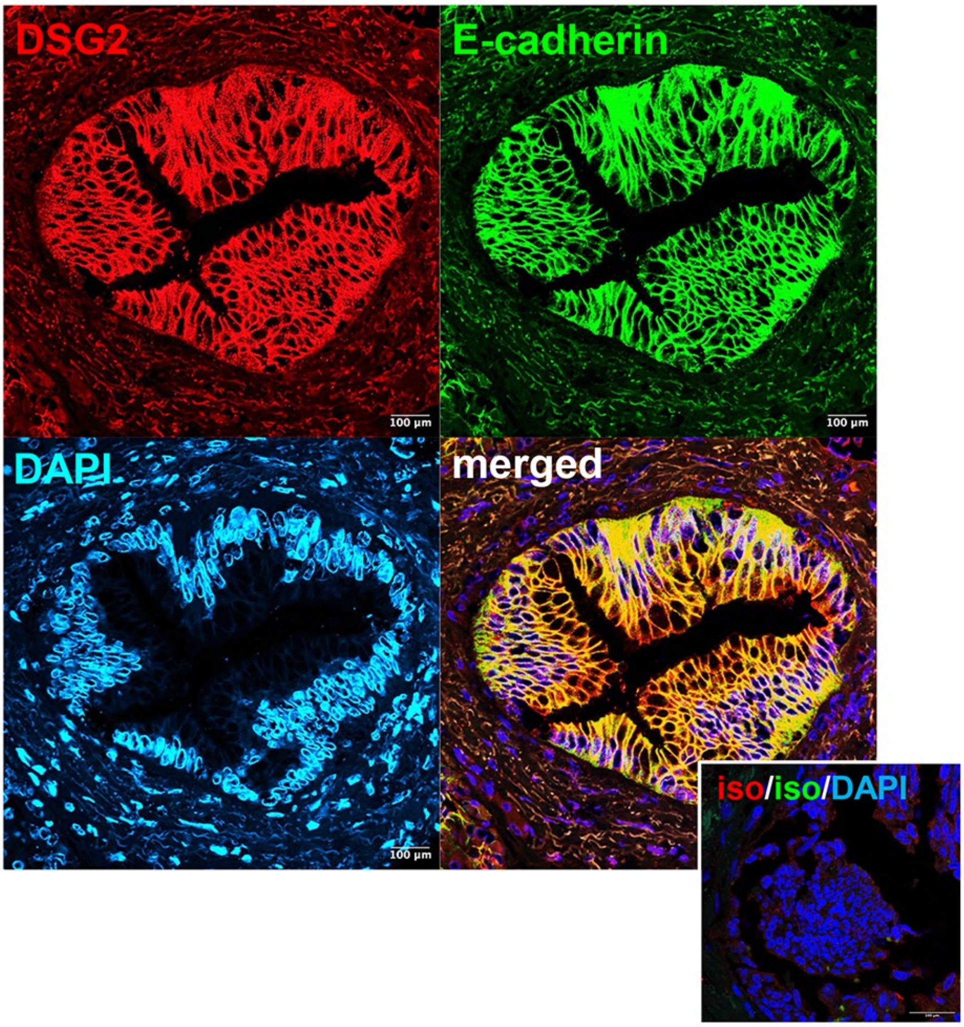

Supplementary Figure S1: DSG2 and E-cadherin expression in healthy human islets

Immunofluorescence confocal microscopy of human pancreas from a healthy body donor stained for DSG2 (red), E-cadherin (green) and nuclei (blue). Insert bottom right is representative of isotype control staining. Scale bar = 100µm.

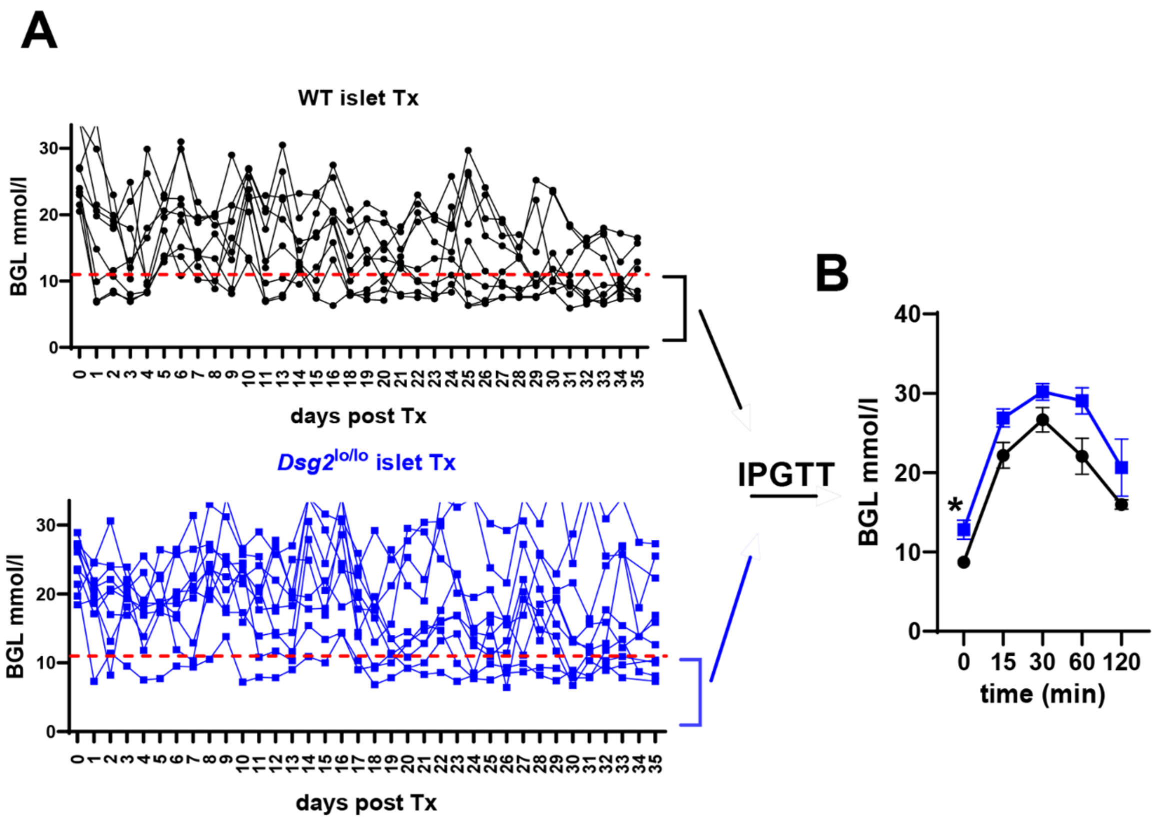

Supplementary Figure S2. Transplantation of islets from WT or *Dsg2$^{lo/lo}$* mice into diabetic WT mice.

Diabetic C57Bl6/N control (WT, n=9) mice were transplanted with marginal islet mass of 200 islets harvested from WT (n=5) or *Dsg2*$^{lo/lo}$ (n=4) mice under the kidney capsule. **A,** BGLs in individual mice were recorded daily for up to 35 days post-transplantation. Black lines represent a single mouse wherein WT islets were transplanted into diabetic WT mice. Blue lines represent a single mouse wherein *Dsg2*$^{lo/lo}$ islets were transplanted into diabetic WT mice. Red dotted line along the x-axis at 11.1 mmol/L indicates euglycaemic glucose levels. **B,** From A, cured mice underwent an IPGTT with 2 mg/kg of glucose administered IP and BGLs determined at 0, 15, 30, 60 and 120 min post injection. Results are mean ± SEM for WT (n=5) and *Dsg2*$^{lo/lo}$ (n=4), *p<0.05 vs WT.

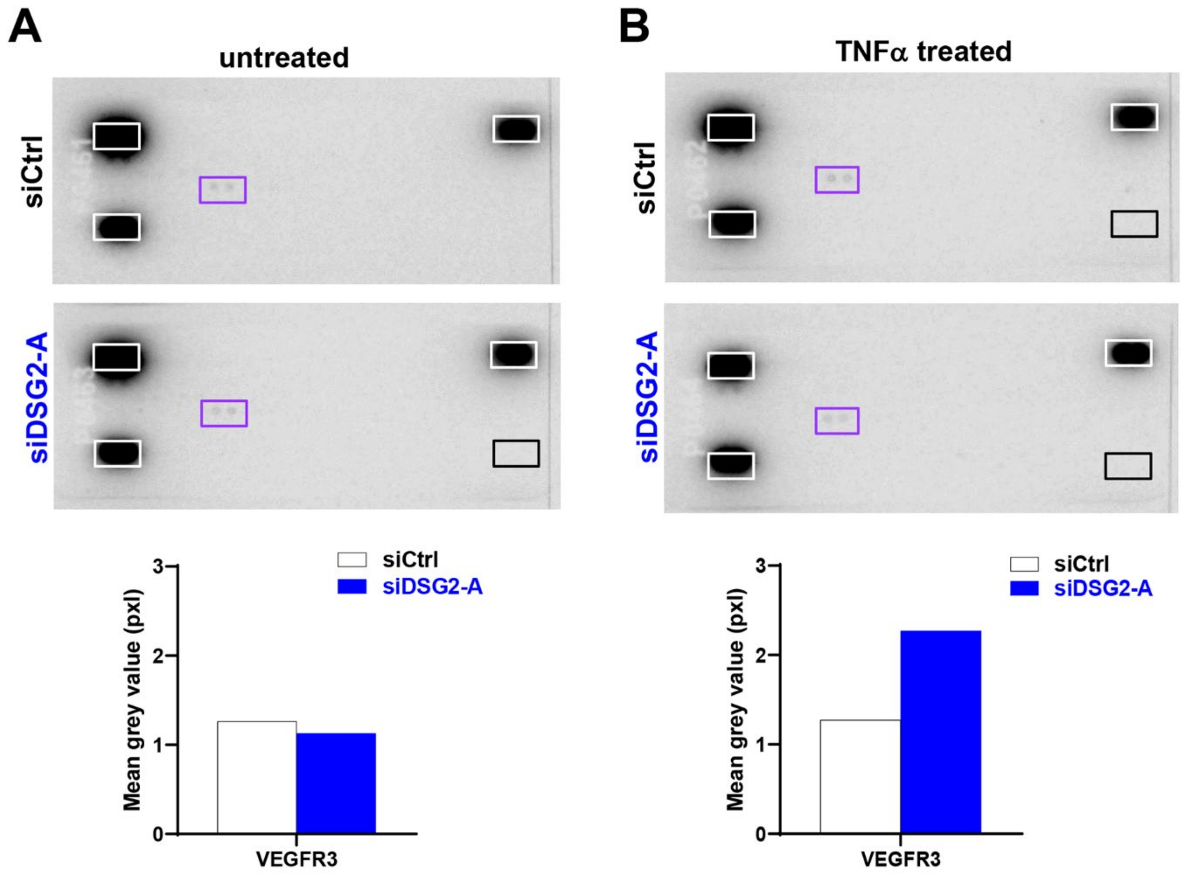

Supplementary Figure S3. Phospho-receptor tyrosine kinase (Phospho-RTK) expression of Beta-TC-6 cells without and with *Dsg2* knockdown.

A Phospho-RTK proteome array of Beta-TC-6 cell lysates without (siCtrl, black) and with *Dsg2* knockdown (siDSG2-A, blue), n=1 experiment. For detectable proteins, mean grey value of duplicate dots was calculated and graphed. White box = positive control, black box = negative control, purple box = VEGFR3. **B** Phospho-RTK proteome array of Beta-TC-6 cell lysates without (siCtrl, black) and with *Dsg2* knockdown (siDSG2-A, blue) following TNFα treatment (100 ng/ml, 24 h), n=1 experiment. For detectable proteins, mean grey value of duplicate dots was calculated and graphed. White box = positive control, black box = negative control, purple box = VEGFR3.
